# Acetylation stabilizes stathmin1 and promotes its activity contributing to gallbladder cancer metastasis

**DOI:** 10.1038/s41420-022-01051-z

**Published:** 2022-05-17

**Authors:** Kun Fan, Xiaojian Ni, Sheng Shen, Zijun Gong, Jiwen Wang, Yanlei Xin, Bohao Zheng, Wentao Sun, Han Liu, Tao Suo, Xiaoling Ni, Houbao Liu

**Affiliations:** 1 Department of General Surgery, Zhongshan Hospital, Fudan University, Shanghai, China; 2 Department of General Surgery, Central Hospital of Xuhui District, Shanghai, China; 3 Biliary Tract Disease Center of Zhongshan Hospital, Fudan University, Shanghai, China; 4 Cancer Center, Zhongshan Hospital, Fudan University, Shanghai, China; 5 Biliary Tract Disease Institute, Fudan University, Shanghai, China

**Keywords:** Metastasis, Translational research

## Abstract

Gallbladder cancer is the most common biliary tract malignant tumor with highly metastatic characters and poor prognosis. However, the underlying mechanism remains unclear. Stathmin1 is ubiquitous phosphoprotein, regulating microtubule stabilization. We identified the acetylation of stahtmin1 at lysine 9 (K9) in gallbladder cancer. K9 acetylation of stathmin1 was reversely regulated by the acetyltransferase PCAF and the deacetylases sirt2. K9 acetylation of stathmin1 inhibited the combining of stathmin1 to E3 ubiquitin ligase RLIM, thereby inhibiting its ubiquitination degradation. Moreover, K9 acetylation also promoted the activity of stahtmin1 interacting and destabilizing microtubule through the inhibition of stathmin1 phosphorylation. K9 acetylated stathmin1 significantly promoted gallbladder cancer cell migration and invasion viability in vitro and lung metastasis in vivo, and indicated poor prognosis of nude mice. IHC assay suggested the positive correlation of high levels of K9 acetylation and stathmin1 expression in gallbladder cancer. Our study revealed that K9 acetylation up-regulated stathmin1 protein stability and microtubule-destabilizing activity to promoted gallbladder cancer metastasis, which provides a potential target for gallbladder cancer therapy.

## Introduction

Gallbladder cancer is the most common biliary tract tumor, accounting for 80–95% of biliary tract tumors, with an annual incidence of almost 10,000 and annual mortality of 3300 [1, 2]. The incidence of gallbladder cancer is more commonly in women than in men [3]. Surgery operation remains the primary therapy, but the recurrence rates are high even though the completed resection [1]. Most patients are diagnosed at advanced stages without effective treatment. Therefore, gallbladder cancer with a high mortality rate and poor median survival of less than one year [2]. It is urgent to reveal the mechanism of gallbladder cancer occurrence and development for therapy.

Stathmin1 is a ubiquitous phosphoprotein, is also the generic element of a protein family including the neural proteins SCG10, SCLIP, RB3 and its two splice variants RB3′and RB3′′ [4], which share a stathmin-like domain including a regulatory region and an interaction region. Four identified phosphorylation sites at ser16/25/38/63 are located at the regulatory region. As a phosphoprotein, stathmin1 integrates a variety of extracellular stimuli including growth and differentiation factors and multiple intracellular signaling pathways through the phosphorylation of four serine sites, and plays a pivotal role in signal transduction [4, 5]. It has reported that CaM kinases II is related to ser16 phosphorylation, ser25 phosphorylation is mainly induced by MAPK, serine 38 is a substrate of p34^cdc2^, ser63 phosphorylation is regulated by PKA [4]. The phosphorylation also plays important roles on stathmin1 activity of regulating microtubule (MT) dynamic progress [4]. The non-phosphorylated stathmin1 interacts with two molecules α/β-tubulin to form a ternary T2S complex, inducing microtubule destabilization [4, 5]. Stathmin1 is also identified as an oncoprotein in many tumors promoting tumor cell proliferation and migration [6–8]. However, the regulation of acetylation modification on stathmin1 is unclear.

To protein acetylation modification, the acetyl group, donated by the metabolite acetyl-coenzyme A, reversibly attached to either the α-amino group of the N-terminus of proteins or to the ε-amino group of lysine residues [9]. Lysine acetylation is an evolutionarily conserved past-transcriptional modification [10], reversibly regulated by acetyltransferases and deacetylases, originally discovered at histones regulating on chromosome structure and gene transcription [11, 12]. The rapid development of mass spectrometric technology contributes the discovery of large numbers of acetylated proteins, including histones and nonhistones, which these proteins cover a broad range of cellular activities including cell cycle, DNA damage check, cytoskeleton organization and metabolism [11–14]. Moreover, the α-tubulin acetylation also establishes the regulation on microtubule stability [15], which indicates the potential acetylation of stathmin1 because of its role of microtubule regulation.

Our study found the K9 acetylation of stathmin1 and revealed the underlying mechanism and its roles on stathmin1, which provides a potential target for gallbladder cancer therapy.

## Results

### Stathmin1 was acetylated at K9

**Fig. 1:**
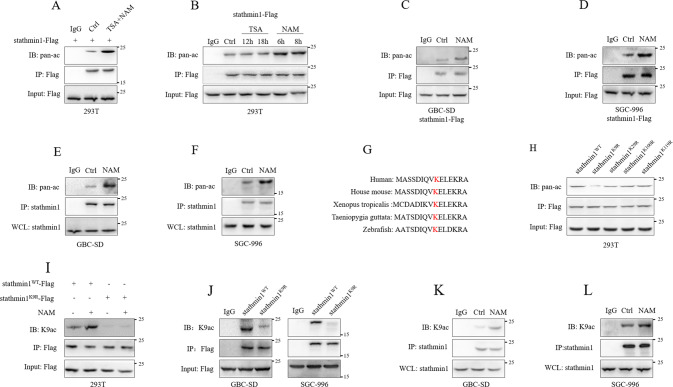
Stathmin1 was acetylated at K9 in GBC. Immunoprecipitation detection of the acetylation of exogenous stathmin1, **A**, **B** after transfection of stathmin1-Flag plasmids to 293 T cells for 48 h with TSA and NAM treatment at indicated time, **C**, **D** in GBC-SD and SGC-996 stable cells with stathmin1 overexpression, **E**, **F** in GBC-SD and SGC-996 cells. **G** The amino acid sequence analysis around K9 in different of species. **H** Immunoprecipitation detection of the acetylation of exogenous stathmin1. Stathmin1^WT^-Flag, stathmin1^K9R^-Flag, stathmin1^K29R^-Flag, stathmin1^K100R^-Flag and stathmin1^K119R^-Flag plasmids were transfected to 293 T cells for 48 h with TSA and NAM treatment. Immunoprecipitation detection of K9 acetylation of exogenous stathmin1 through K9ac specific antibody, **I** after transfection of stathmin1^WT^-Flag and stathmin1^K9R^-Flag plasmids to 293 T cells for 48 h with NAM treatment, **J** in GBC-SD and SGC-996 stable cells overexpressing stathmin1, **K**, **L** in GBC-SD and SGC-996 cells. Results were shown as means ± s.d from at least three independent experiments.

Rapid development of mass spectrometric technology identifies a large number of acetylated proteins [11]. Public repositories such as PhosphoSitePlus database show more than 24000 lysine acetylation sites in human cells [16]. A study of protein mass spectrum indicated several possible lysine acetylation sites of stathmin1 [17]. To ensure acetylation modification of stathmin1, we transfected stathmin1-Flag plasmids to 293 T cells with the treatment of nicotinamide (NAM), an inhibitor of SIRT deacetylases family and trichostatin A (TSA), an inhibitor of histone deacetylase (HDAC) class I and class II [12, 13, 18]. Immunoprecipitation results indicated the possible acetylation of stathmin1, and treatment of NAM and TSA up-regulated the acetylation level of stathmn1 (Fig. 1A). It was NAM, not TSA that caused the up-regulation of stathmin1 acetylation through the separate treatment (Fig. 1B). We next established GBC-SD and SGC-996 stable cells with stathmin1 overexpression. Immunoprecipitation detection also confirmed that acetylation of stathmin1 was up-regulated by NAM treatment in GBC-SD and SGC-996 stable cells (Fig. 1C, D). We next employed stathmin1 antibody to pull down endogenous stathmin1 in GBC-SD and SGC-996 cells and detected significant acetylation of endogenous stathmin1 up-regulated by NAM treatment (Fig. 1E, F), which further confirmed the acetylation of stathmin1. Protein mass spectrum data indicated several lysine acetylation sites at K9, K100 and K119 of stathmin1 [17]. Another study showed lysine methylation at K29 of stathmin1 [19]. To explore the acetylated lysine of stathmin1, we analyzed the amino acid sequences around K9, K29, K100, and K119 sites and found the amino acid sequence around K9 was conserved (Fig. 1G). Moreover, K9 acetylation was also identified through the mass spectrum (Fig. S1). Next, the plasmids with K9R, K29R, K100R and K119R mutations were transfected to 293 T cells. Results showed K9R mutation significantly reduced the acetylation of stathmin1, indicating the acetylation at K9 (Fig. 1H). We next generated specific antibody recognizing the acetylated K9 (K9ac) of stathmin1. After transfection of stathmin1^WT^-Flag and stathmin1^K9R^-Flag plasmids with NAM treatment, the detection through K9ac antibody verified that K9 acetylation of mutant stathmin1 was reduced compared with wild type stathmin1. NAM treatment could up-regulate the K9 acetylation of wild type stathmin1, but not affect mutant stathmin1 (Fig. 1I). We also established GBC-SD and SGC-996 stable cells with K9R mutant stathmin1. K9 acetylation of mutant stathmin1 was lower than wild type stathmin1 (Fig. 1J). K9 acetylation of endogenous stahtmin1 was also upregulated by NAM in GBC-SD and SGC-996 cells (Fig. 1K, L). These results confirmed the K9 acetylation of stahtmin1 in gallbladder cancer cells.

### K9 acetylation inhibited ubiquitination degradation of stathmin1

**Fig. 2:**
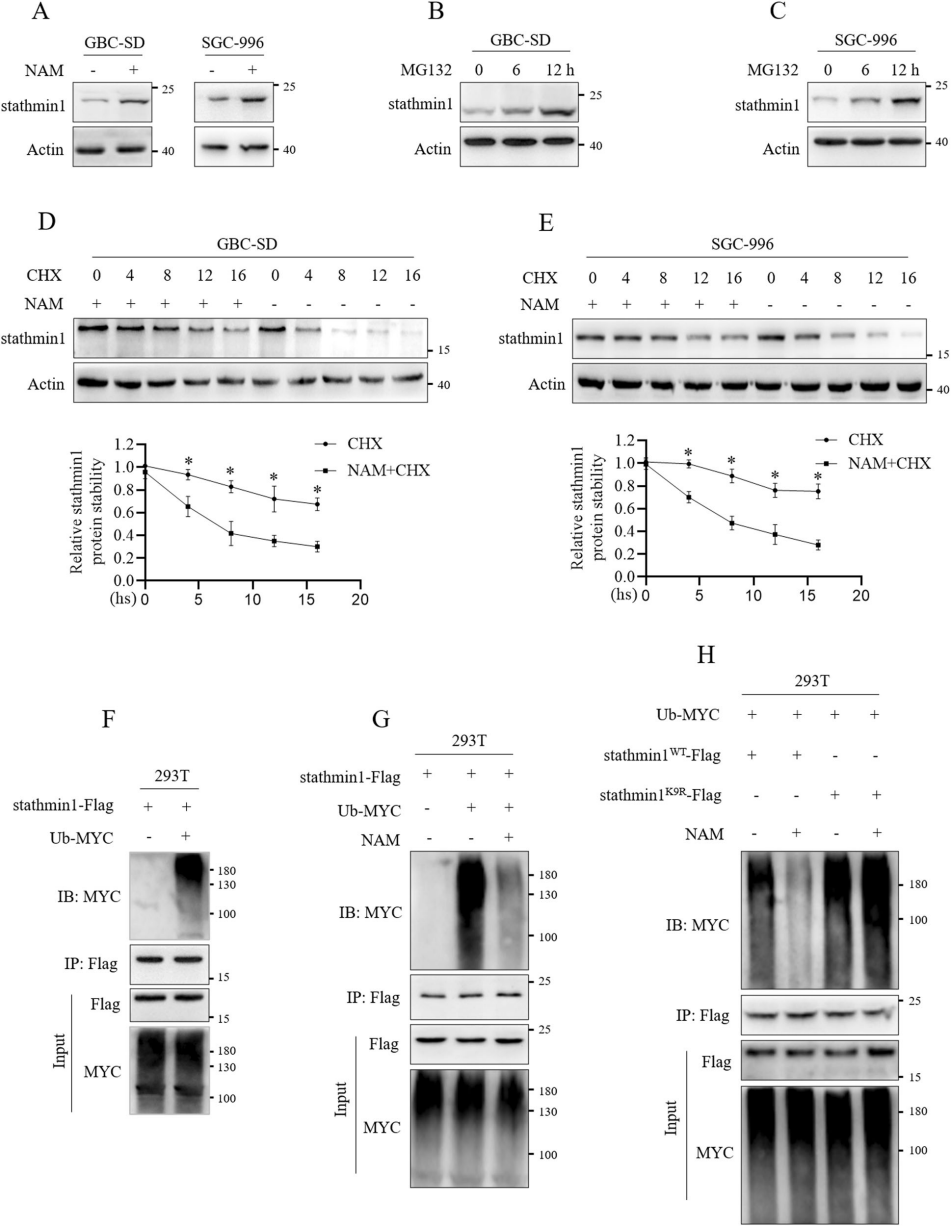
K9 acetylation inhibited the ubiquitination degradation to promote stathmin1 protein stability. Stathmin1 protein level was analyzed through western blot in GBC-SD and SGC-996 cells, **A** after NAM treatment, **B**, **C** after MG132 treatment at indicated time. **D**, **E** Western blot analysis of stathmin1 protein stability after NAM and CHX treatment. Immunoprecipitation analysis of ubiquitination of stathmin1, **F** after transfection of stathmin1-Flag and Ub-MYC plasmids to 293 T cells for 48 h, **G** with NAM treatment after plasmids transfection for 48 h, **H** with Stathmin1^WT^-Flag, Stathmin1^K9R^-Flag plasmids transfection and NAM treatment. Results were shown as means ± s.d from three independent experiments, *p < 0.05.

Protein acetylation plays important roles in regulating protein stability and enzyme activity [11]. The roles of K9 acetylation on stathmin1 remain unclear. We firstly detected stathmin1 protein stability. After NAM treatment, stathmin1 protein level was increased (Fig. 2A), which indicated the possible regulation of K9 acetylation on stathmin1 protein stability. MG132 treatment significantly up-regulated stathmin1 protein level (Fig. 2B, C), which suggested stathmin1 was degraded through ubiquitination-proteasome pathway. We used NAM and CHX (cycloheximide) to treat GBC-SD and SGC-996 cells and found the treatment of CHX and NAM could inhibit stathmin1 protein degradation compared with only CHX treatment (Fig. 2D, E). Therefore, we speculated that K9 acetylation possibly inhibited stathmin1 ubiquitination degradation. Results of transfection of stathmin1-Flag and Ub-MYC plasmids showed the ubiquitination degradation of stathmin1 (Fig. 2F), which was significantly inhibited NAM treatment (Fig. 2G). Moreover, we transfected stathmin1^WT^-Flag, stathmin1^K9R^-Flag and Ub-MYC plasmids with NAM treatment and verified that mutant stathmin1 showed more drastic degradation compared with wild type stathmin1, NAM treatment showed inhibition on wild type stathmin1, not mutant stathmin1 (Fig. 2H). These results suggested that K9 acetylation inhibited ubiquitination degradation to promote the protein stability of stathmin1.

### K9 acetylation inhibited the phosphorylation of stathmin1 to promote the combination with tubulin

**Fig. 3:**
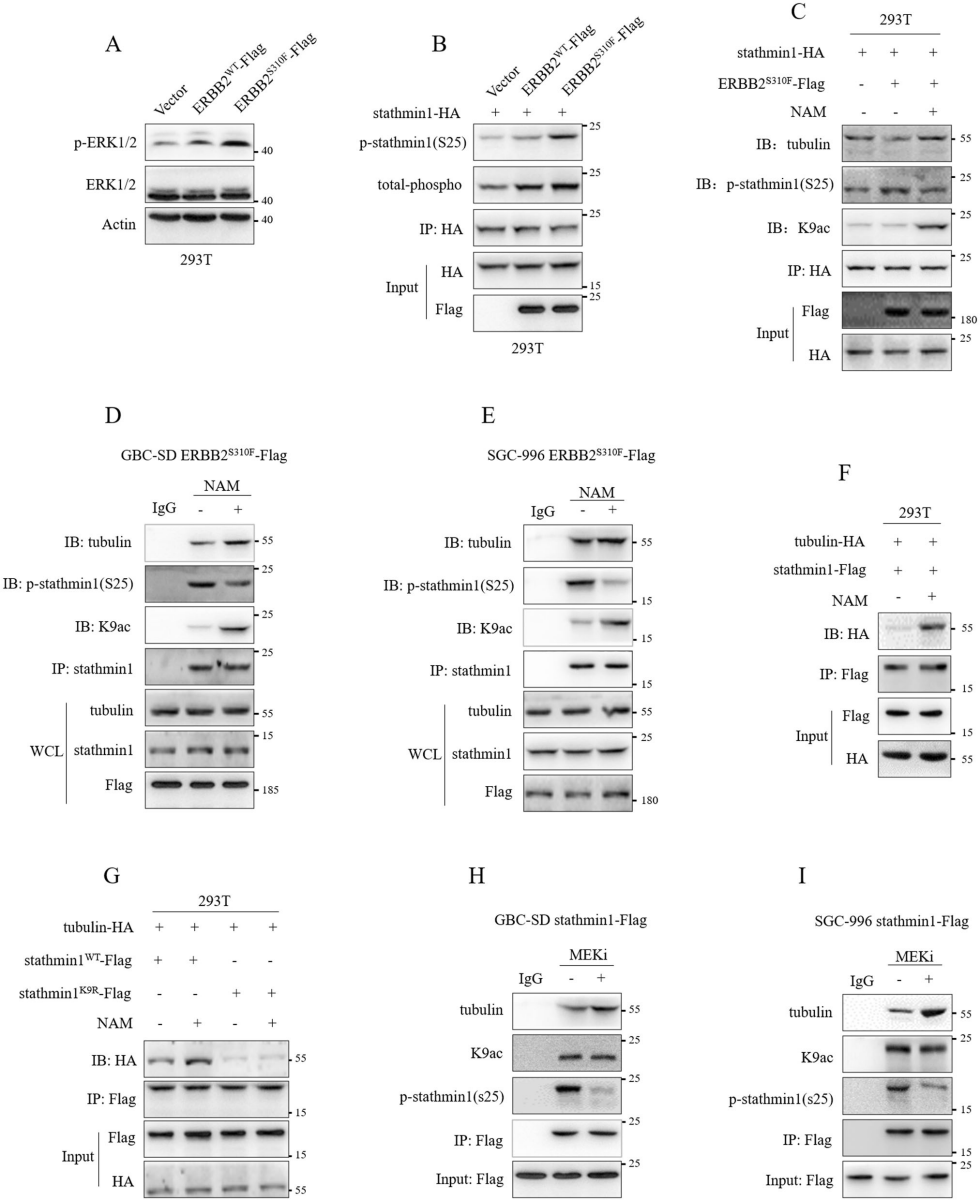
K9 acetylation promoted the combination of stathmin1 and tubulin through inhibiting the phosphorylation of stathmin1. **A** Western blot detection of the ERK1/2 phosphorylation. The ERBB2^WT^-Flag, ERBB2^S310F^-Flag and vector plasmids were transfected to 293 T cells for 48 h. **B** Immunoprecipitation detection of the phosphorylation of stathmin1. The ERBB2^WT^-Flag, ERBB2^S310F^-Flag and vector plasmids were co-transfected with stathmin1-HA plasmids to 293 T cells for 48 h. Co-immunoprecipitation detection of the interaction of stathmin1 and tubulin, **C** after transfection of stathmn1-HA and ERBB2^S310F^-Flag plasmids to 293 T cells with NAM treatment, **D**, **E** in the ERBB2^S310F^ overexpressed GBC-SD and SGC-996 stable cells with NAM treatment, **F** after transfection of stathmin1-Flag and tubulin-HA plasmids to 293 T cells for 48 h with NAM treatment, **G** after transfection of stathmin1^WT^-Flag, stathmin1^K9R^-Flag and tubulin-HA plasmids to 293 T cells for 48 h with NAM treatment, **H**, **I** in GBC-SD and SGC-996 stable cells with stathmin1 overexpression treated by NAM. Results were shown as means ± s.d, represented three independent experiments.

Stathmin1 combines with α/β-tubulin to regulate microtubule stabilization in a phosphorylation-dependent way and its phosphorylation at ser 25 is associated to MAPK pathway [4, 5]. Whether K9 acetylation of stathmin1 had an effect on stathmin1 activity. Our previous study has verified that EBBB2 S310F mutation could activate MAPK/ERK pathway [2]. We transfected ERBB2^WT^-Flag and ERBB2^S310F^-Flag plasmids to 293 T cells and verified that mutant ERBB2 showed more significant activation on MAPK/ERK pathway compared with wild type ERBB2 (Fig. 3A). The transfection of stathmin1-HA, ERBB2^WT^-Flag and ERBB2^S310F^-Flag plasmids verified that the dramatical increase of ser25 phosphorylation in the mutant ERBB2 group (Fig. 3B). Next, the transfection of stathmin1-HA and ERBB2^S310F^-Flag plasmids to 293 T cells with NAM treatment showed that mutant ERBB2 could decrease the combination with tubulin through the increase of ser25 phosphorylation. However, NAM treatment promoted K9 acetylation of stathmin1 through decreasing the ser25 phosphorylation, which promoted the interaction of stathmin1 with tubulin (Fig. 3C). Through GBC-SD and SGC-996 stable cells with ERBB2 S310F mutation, we further verified that NAM treatment increased K9 acetylation of stathmin1 to inhibit the ser25 phosphorylation and combination with tubulin (Fig. 3D, E). Moreover, transfection of stathmin1-Flag and tubulin-HA plasmids also showed the increased combination of sathmin1 and tubulin by NAM treatment (Fig. 3F). We next transfected tubulin-HA, stathmin1^WT^-Flag and stathmin1^K9R^-Flag plasmids to 293 T cells with NAM treatment. The combination with tubulin of mutant stathmin1 was significantly decreased, NAM treatment could promote the combination of tubulin and wild type stathmin1, but not affect the mutant stathmin1 (Fig. 3G). We also employed binimetinib treatment, a MEK inhibitor, in GBC-SD and SGC-996 stable cells with stathmin1 overexpression and confirmed the treatment of MEK inhibitor could decrease stathmin1 ser25 phosphorylation and the combination with tubulin, but not affect K9 acetylation (Fig. 3H, I). These results suggested that K9 promoted stathmin1 interacting with tubulin through decreasing the ser25 phosphorylation of stathmin1.

### PCAF mediated K9 acetylation of stathmin1

**Fig. 4:**
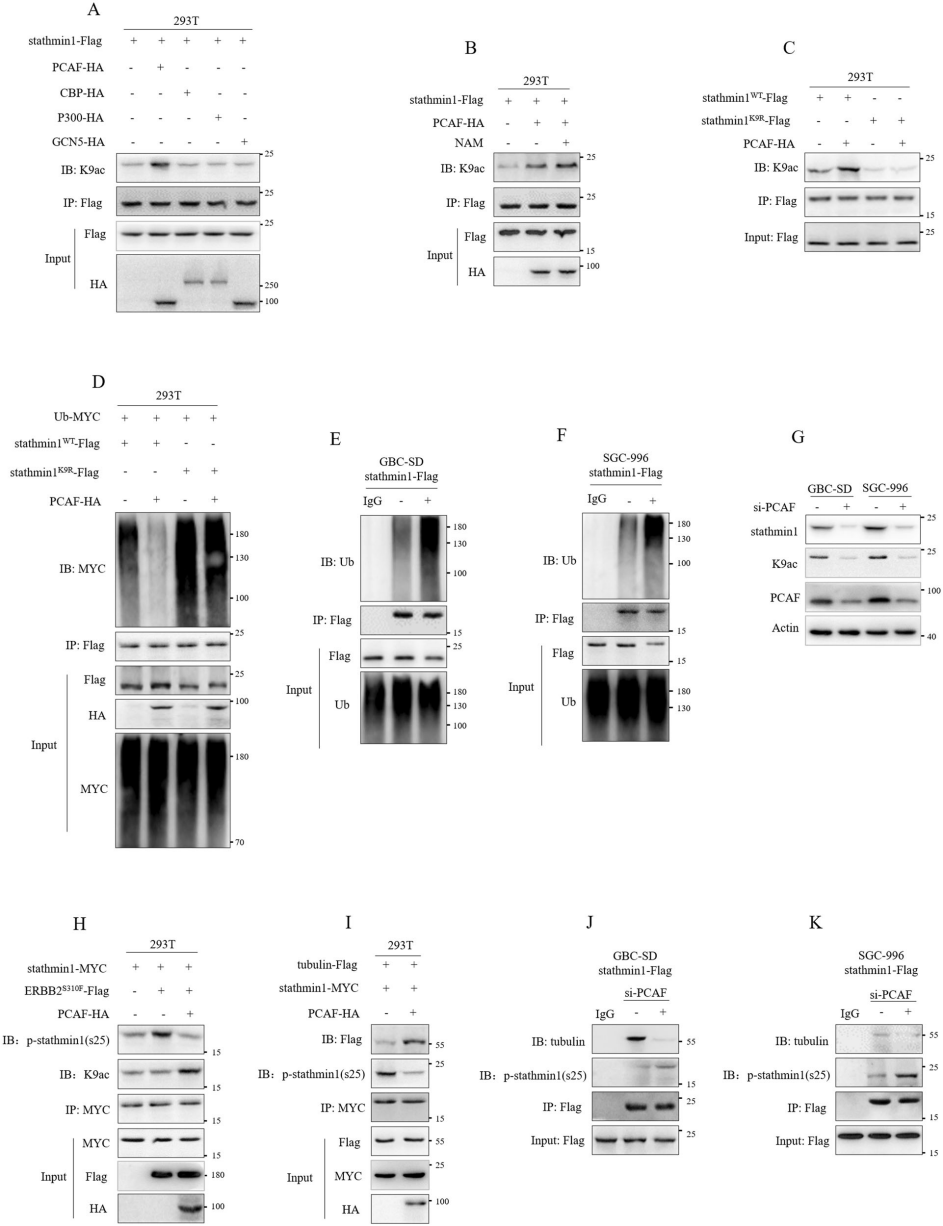
PCAF mediated the K9 acetylation of stathmin1. Immunoprecipitation detection of K9 acetylation of stathmin1, **A** after co-transfection of plasmids of PCAF-HA, CBP-HA, P300-HA and GCN5-HA with stathmin1-Flag plasmids to 293 T cells for 48 h, **B** after transfection of plasmids of stathmin1-Flag and PCAF-HA to 293 T cells with NAM treatment, **C** after transfection of plasmids of stathmin1^WT^-Flag, stathmin1^K9R^-Flag and PCAF-HA to 293 T cells for 48 h, **D** after transfection of plasmids of stathmin1^WT^-Flag, stathmin1^K9R^-Flag, PCAF-HA and Ub-MYC to 293 T cells for 48 h, **E**, **F** in GBC-SD and SGC-996 stable cells overexpressing stathmin1. (**G**) Western blot detection of stathmin1 protein level after PCAF knockdown by targeting siRNA, non-targeting siRNA as control. Immunoprecipitation detection of the phosphorylation and acetylation levels of stathmin1, **H** after transfection of plasmids of stathmin1-MYC, ERBB2^S310F^-Flag and PCAF-HA to 293 T cells for 48 h, **I** after transfection of plasmids of stathmin1-MYC, Tubulin-Flag and PCAF-HA to 293 T cells for 48 h, **J**, K in GBC-SD and SGC-996 stable cells after PCAF knockdown. Results were shown as means ± s.d with at least three independent experiments.

Lysine acetylation was carried out by acetyltransferases. After the identification of K9 acetylation of stathmin1, we next explored the possible acetyltransferases. According to several previous studies [12–14], four acetyltransferases including p300 (E1A binding protein), CBP (CREB binding protein), PCAF (p300/CBP-associated factor, or KAT2B) and GCN5 (KAT2A) were detected. We co-transfected stathmin1-Flag, PCAF-HA, CBP-HA and P300-HA plasmids to 293 T cells, then stathmin1 was pulled down and K9 acetylation was detected. Results showed that PCAF transfection significantly increased K9 acetylation of stathmin1 (Fig. 4A), which was further increased by NAM (Fig. 4B). Co-transfection of wild-type stathmin1, K9R mutant stathmin1 and PCAF-HA plasmids showed PCAF could up-regulate K9 acetylation of wild type stathmin1, but not affect acetylation of mutant stathmin1, and mutant stathmin1 also showed lower K9 acetylation level than wild type stathmin1 (Fig. 4C). The decreased K9 acetylation of stathmin1 by PCAF knockdown was also verified in GBC-SD and SGC-996 stable cells (Fig. 4H). Therefore, PCAF mediated stathmin1 K9 acetylation. We next analyzed the role of PCAF on ubiquitination degradation of stathmin1. We co-transfected stathmin1^WT^-Flag, stathmin1^K9R^-Flag, PCAF-HA and Ub-MYC plasmids and verified that PCAF significantly inhibited ubiquitination degradation of wild type stathmin1, but not affect mutant stathmin1 (Fig. 4D). PCAF knockdown caused significant increase of stathmin1 degradation in GBC-SD and SGC-996 stable cells (Fig. 4E, F). Moreover, the levels of K9 acetylation and protein of stathmin1 were decreased after PCAF knockdown in GBC cells (Fig. 4G). These results suggested that PCAF could inhibit stathmin1 ubiquitination degradation to increase stathmin1 protein stability through acetylating stathmin1. We next analyzed the role of PCAF on stathmin1 activity. The co-transfection of stathmin1-MYC, ERBB2^S310F^-Flag and PCAF-HA plasmids showed that PCAF could promote K9 acetylation of stathmin1 to decrease ser25 phosphorylation induced by mutant ERBB2 (Fig. 4H). Co-transfection of tubulin-Flag, stathmin1-MYC and PCAF-HA plasmids showed that PCAF also promoted the combination of stathmin1 and tubulin (Fig. 4I). However, knockdown PCAF by siRNA significantly decreased the interaction of stathmin1 and tubulin in GBC-SD and SGC-996 stable cells (Fig. 4J, K). These results suggested that PCAF mediating K9 acetylation of stathmin1 promoted its combination stathmin1 with tubulin through inhibiting the phosphorylation of stathmin1.

### Sirt2 mediated the K9 deacetylation of stathmin1

**Fig. 5:**
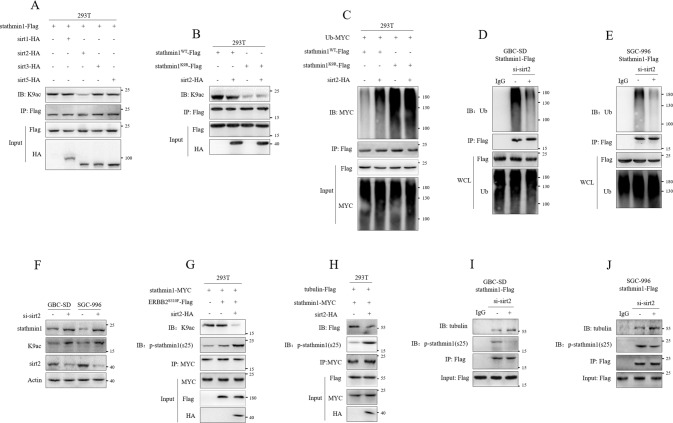
Sirt2 mediated the deacetylation of stathmin1. Immunoprecipitation detection of K9 acetylation of stathmin1, **A** after co-transfection of plasmids of sirt1-HA, sirt2-HA, sirt3-HA and sirt5-HA with stathmin1-Flag to 293 T cells for 48 h, **B** after transfection of plasmids of stathmin1^WT^-Flag, stathmin1^K9R^-Flag and sirt2-HA to 293 T cells for 48 h, **C** after transfection of plasmids of stathmin1^WT^-Flag, stathmin1^K9R^-Flag, sirt2-HA and Ub-MYC to 293 T cells for 48 h. **D**, **E** Immunoprecipitation assay of ubiquitination of stathmin1 in GBC-SD and SGC-996 stable cells with sirt2 knockdown by targeting siRNA. **F** Western blot detection of stathmin1 protein and K9 acetylation levels. The siRNA targeting sirt2 was transfected to GBC-SD and SGC-996 cells for 48 h, nontargeting siRNA as control. **G** Immunoprecipitation detection of the phosphorylation and acetylation levels of stathmin1. The plasmids of stathmin1-MYC, ERBB2^S310F^-Flag and sirt2-HA were transfected to 293 T cells for 48 h. Co-immunoprecipitation detection of the interaction of stathmin1 and tubulin, **H** after transfection of plasmids of stathmin1-MYC, Tubulin-Flag and sirt2-HA to 293 T cells for 48 h, **I**, **J** in GBC-SD and SGC-996 stable cells after sirt2 knockdown. Results shown as means ± s.d represented at least three independent experiments.

NAM treatment promoting K9 acetylation of stathmin1 suggested that the deacetylation of stathmin1 was regulated by SIRT family deacetylase. According to previous study and the localization of SIRT family members [14, 18], we co-transfected sirt1-HA, sirt2-HA, sirt3-HA and sirt5-HA with stathmin1-Flag plasmids to 293 T cells. Results showed that sirt2 significantly decreased K9 acetylation of stathmin1 (Fig. 5A), which indicated sirt2 mediated the deacetylation of stathmin1. Therefore, we next transfected the stathmin1^WT^-Flag, stathmin1^K9R^-Flag, sirt2-HA plasmids to 293 T cells. Results showed that sirt2 decreased the K9ac of wild type stathmin1, but not affect mutant stathmin1(Fig. 5B). These suggested that sirt2 mediated stathmmin1 K9 deacetylation. We next analyzed the role of sirt2 on ubiquitination degradation of stathmin1. We transfected Ub-MYC, stathmin1^WT^-Flag, stathmin1^K9R^-Flag and sirt2-HA plasmids to 293 T cells. Sirt2 significantly increased ubiquitination degradation of wild type stathmin1, but had no effect on mutant stathmin1, and the degradation of mutant stathmin1 also was more intense than wild type stathmin1 (Fig. 5C). In GBC-SD and SCG-996 stable cells, the ubiquitination degradation of stathmin1 was inhibited by sirt2 knockdown (Fig. 5D, E). Moreover, we knocked down endogenous sirt2 in GBC-SD and SGC-996 cells and found that both stathmin1 protein and K9 acetylation levels were increased (Fig. 5F). These results indicated that sirt2 mediating K9 deacetylation of stathmin1 promoted the ubiquitination degradation with decreased protein stability. We next analyzed the role of sirt2 on the activity of stathmin1 destabilizing microtubule. Co-transfection of stathmin1-MYC, ERBB2^S310F^-Flag and sirt2-HA plasmids to 293 T cells showed that sirt2 decreased K9 acetylation of stathmin1, but increased ser25 phosphorylation of stathmin1 (Fig. 5G). We next transfected tubulin-Flag, stathmin1-MYC and sirt2-HA plasmids, verified that sirt2 could inhibit stathmin1 combining with tubulin (Fig. 5H). The combination stathmin1 and tubulin was increased by sirt2 knockdown in GBC-SD and SGC-996 stable cells (Fig. 5I, J). These results suggested that sirt2 mediating K9 deacetylation of stathmin1 promoted ubiquitination degradation of stathmin1, increased the ser25 phosphorylation to inhibit the combination of stathmin1 with tubulin.

### K9 acetylation of stathmin1 inhibited the combination with RLIM decreasing stathmin1 degradation

**Fig. 6:**
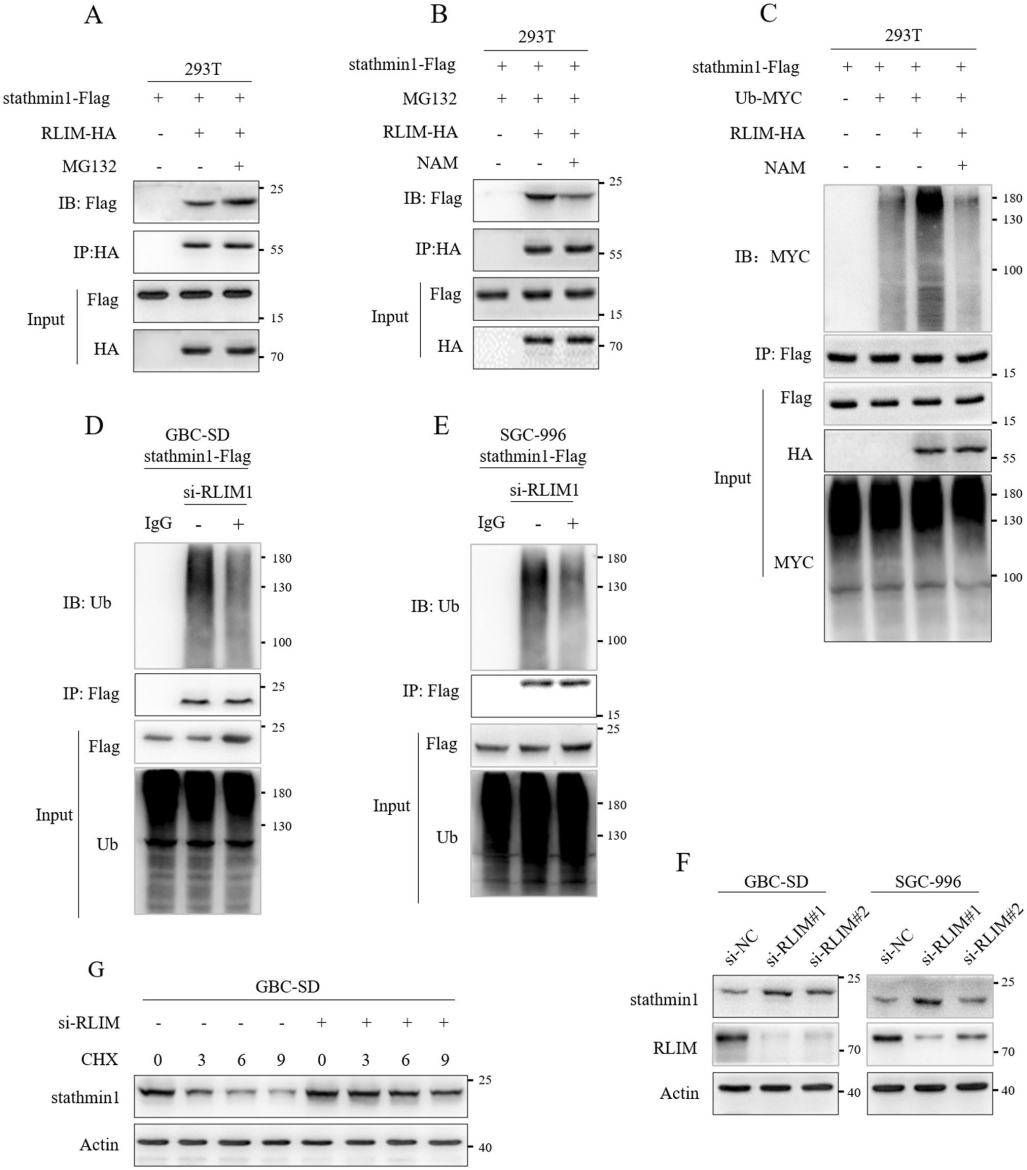
K9 acetylation inhibited the combination of stathmin1 with RLIM. Immunoprecipitation detection of the interaction of stathmin1 and RLIM, **A** after transfection of plasmids of stathmin1-Flag and RLIM-HA to 293 T cells with MG132 treatment, **B** after transfection of plasmids of stathmin1-Flag and RLIM-HA to 293 T cells with MG132 and NAM treatment, **C** after transfection of plasmids of stathmin1-Flag, RLIM-HA and Ub-MYC to 293 T cells with NAM treatment, **D**, **E** in GBC-SD and SGC-996 stable cells with stathmin1 overexpression. **F** Western blot detection of stathmin1 protein level. The siRNAs targeting RLIM were transfected to GBC-SD and SGC-996 cells for 48 h, nontargeting siRNA as negative control. **G** Western blot detection of stathmin1 protein stability. GBC-SD cells were transfected by the siRNA targeting RLIM for 48 h, and treated by CHX. All results shown as means ± s.d represented at least three independent experiments.

RLIM was identified as an E3 ubiquitin ligase regulating the stability of stathmin1 protein in two studies [20, 21]. MG132 treatment increased the combination of stathmin1 and RLIM (Fig. 6A). We next transfected stathmin1-Flag and RLIM-HA plasmids with MG132 and NAM treatment. NAM treatment significantly inhibited the combination of stathmin1 and RLIM (Fig. 6B). We further co-transfected stathmin1-Flag, Ub-MYC and RLIM-HA plasmids. RLIM promoted the ubiquitination degradation of stathmin1, which was inhibited by NAM treatment (Fig. 6C). NAM treatment also decreased the combination of stathmin1 and RLIM in GBC-SD and SGC-996 stable cells (Fig. 6D, E). Moreover, knockdown of RLIM through siRNA increased stathmin1 protein level in GBC-SD and SGC-996 cells (Fig. 6F). We employed CHX and siRNA targeting RLIM to treat GBC-SD cells, the stathmin1 protein stability was significantly increased (Fig. 6G). These indicated that K9 acetylation inhibited stathmin1 combining with RLIM, thereby suppressed the degradation of stathmin1.

### K9 acetylation of stathmin1 promoted gallbladder carcinoma metastasis

**Fig. 7:**
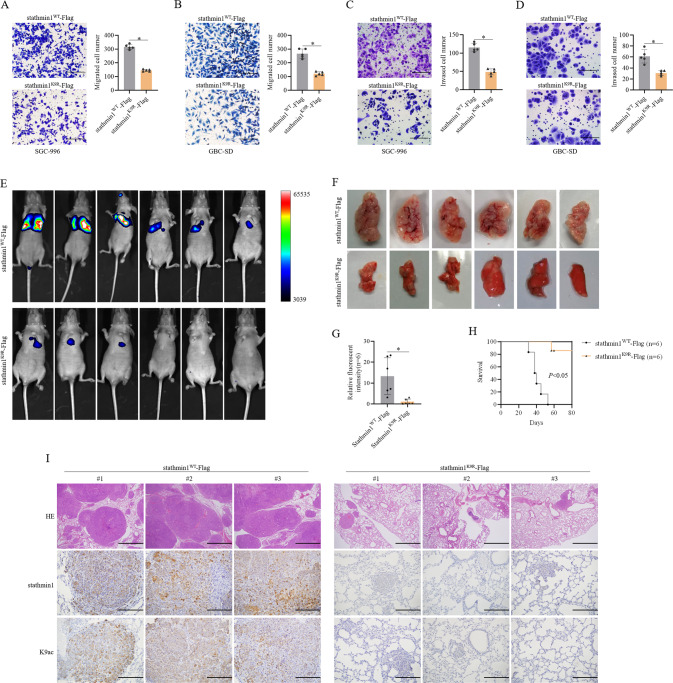
K9 acetylated stathmin1 promoted GBC metastasis. **A**, **B** Cell migration assay in SGC-996 and GBC-SD stable cells in vitro. **C**, **D** Cell invasion assay through coated transwell in SGC-996 and GBC-SD stable cells in vitro. SGC-996 and GBC-SD cells were employed to establish stable cells overexpressing wild type stathmin1 and K9R mutant stathmin1 with luciferase marker. The bar graph of transwell results shown as means ± s.d of five random fields with at least three independent experiments. Scale bar: 200 μm. **E** Live imaging detection of luciferase expression from pulmonary tumors in vivo. SGC-996 stable cells were seed to mice through tail intravenous injection for establishing the model of gallbladder cancer lung metastasis. The metastatic tumors were monitored every two weeks through Tanon ABL X5 Live Animal Imaging System. **F** Lungs images of both wild type stathmin1 and mutant stathmin1 groups resected from gallbladder cancer lung metastasis mice model. **G** Quantification of luciferase expression of pulmonary tumors. **H** Survival analysis of gallbladder cancer lung metastasis mice. **I** Upper: HE staining analysis of lung tissues. Lower: IHC analysis of K9 acetylation of stathmin1 and its protein levels in lung tissues of mice. GBC lung metastasis results in vivo shown as means ± s.d with six mice for each group. Scale bar: 200 μm.

We next analyzed the role of K9 acetylation of stathmin1 on gallbladder carcinoma metastasis. We firstly employed transwell for migration assay and coated transwell for invasion assay in vitro. The SGC-996 and GBC-SD stable cells overexpressing wild-type stathmin1 showed more migrated cells than mutant stathmin1 cells (Fig. 7A, B). Moreover, the number of invaded cells of wild-type stathmin1 was higher than mutant stathmin1 cells (Fig. 7C, D). We next detected the role of K9 acetylation on gallbladder carcinoma metastasis in vivo. SGC-996 stable cells were injected to nude mice via tail vein, then monitored every 2 weeks. Live imaging showed stronger fluorescent signaling of tumor in lung in wild-type stathmin1 groups than that in mutant stathmin1groups (Fig. 7E and G). Moreover, the growth of metastasized tumor was quicker of wild-type stathmin1 groups than mutant stathmin1 groups (Fig. 7F). The mice of wild-type stathmin1 groups had poorer survival compared with mutant stathmin1 groups (Fig. 7H). Hematoxylin-eosin (HE) staining results showed stronger tumor invasion in mice lung of wild-type stathmin1 groups than mutant stathmin1 groups (Fig. 7I). These results suggested that K9 acetylation of stathmin1 promoted gallbladder carcinoma metastasis.

### GBC tissue overexpressed stathmin1 with high K9 acetylation level

**Fig. 8:**
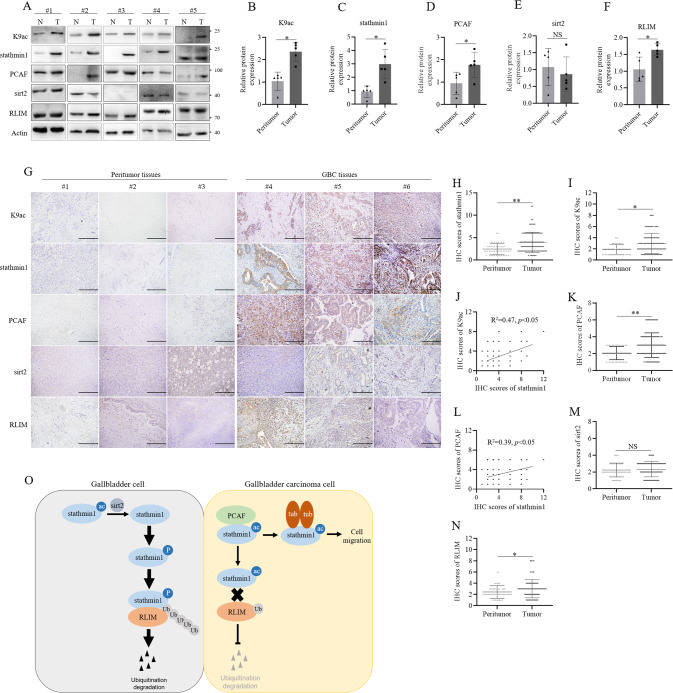
K9 acetylation of stathmin1 were overexpressed in GBC tissues. **A** Western blot analysis of K9 acetylation of stathmin1 and the protein levels in five pairs of GBC and peritumor tissues. **B**–**F** Quantification of K9 acetylation of stathmin1, stathmin1, PCAF, sirt2 and RLIM levels in five pairs of GBC and peritumor tissues. The bar graph represented mean ± sd of five pairs of samples, *p < 0.05. **G** IHC analysis of K9 acetylation of stathmin1, stathmin1, PCAF and sirt2 and RLIM levels in 110 GBC samples and 50 peritumor tissues. Scale bar: 200 μm. **H, I, K, M, N** Quantification of K9 acetylation of stathmin1, stathmin1, PCAF, sirt2 and RLIM levels in GBC and peritumor tissues of IHC results. **J** and **L** Correlation analysis of K9 acetylation and stathmin1 protein levels, PCAF and stathmin1 protein levels in 110 GBC samples. The scatter graph represented mean ± sd, *p < 0.05. **O** A schematic diagram of K9 acetylation of stathmin1 promoting gallbladder cancer metastasis.

To further confirm the role of K9 acetylation of stathmin1 in GBC, five pairs GBC samples with adjacent tissues were detected through western blot. Stathmin1 and K9 acetylation levels were significantly higher in tumor tissues than adjacent tissues (Fig. 8A-C). Likewise, PCAF and RLIM also showed higher expression in tumor tissues and adjacent tissues (Fig. 8A, D, F). However, sirt2 expression did not show significant difference in tumor tissues compared with adjacent tissues (Fig. 8A, E). We next analyzed K9 acetylation of stathmin1 in 110 GBC tissues and 50 adjacent tissues through IHC assay. Consistent to western blot results, IHC results showed the levels of K9 acetylation and stathmin1 in GBC tissues were higher in tumor tissues than adjacent tissues, and significant correlation in tumor tissues (Fig. 8G-J). PCAF showed similar higher expression in tumor tissues compared with adjacent tissues, which was correlated to stathmin1 level (Fig. 8G, K, L). Sirt2 expression did not show significant difference in tumor tissues and adjacent tissues (Fig. 8G, M). RLIM showed slightly high expression in tumor tissues compared with adjacent tissues (Fig. 8G, N). Overexpression of K9 acetylation and stathmin1 in GBC indicated the important roles in clinical.

## Discussion

Most patients with gallbladder cancer are diagnosed at advanced stages without effective therapy. Our previous study has identified a potential candidate of stathmin1 in GBC through two-dimensional electrophoresis combined with mass spectrometry [8, 22]. In this study, we established the roles of stathmin1 promoting gallbladder carcinoma metastasis.

Several studies have verified that stathmin1 transcription was regulated by wild type and mutant p53 [23, 24]. Two SNPs identified in the canine stathmin1 promoter region could affect transcription factor binding [25]. MiR-34a downregulates stathmin1 by directly binding to its 3’-UTR [26]. Interleukin-10 (IL-10) also plays important roles in regulating stathmin1 expression [27]. Several studies have identified four phosphorylated sites of stathmin1 regulating its activity [4, 5]. Besides that, few studies are related to post-translation of stathmin1. The mass spectrum data indicated potential acetylation of stathmin1 [17]. Whether stathmin1 was regulated by acetylation at post-translation level. We employed TSA and NAM treatment according to previous studies [12–14] to confirm the acetylation modification of stathmin1. Moreover, it is NAM treatment, not TSA treatment that upregulated acetylation level of stathmin1. We next would like to confirm the acetylated sites of stathmin1. The mass spectrum data indicated the possible modification at K9, K29, K100 and K119 of stathmin1 [17, 19]. Amino sequence around K9 was more conserved that other sites. Moreover, K9 acetylation of stathmin1 was further identified through site-mutation and K9ac specific antibody in GBC cells.

Now that K9 acetylation of stathmin1 was verified, we next explored the roles of K9 acetylation on stathmin1. Previous studies have reported the regulation of acetylation on protein stability, localization and enzyme activity [11]. The further exploration verified that K9 acetylation enhanced stathmin1 protein stability through inhibiting ubiquitination degradation. These also indicated the correlation of K9 acetylation and stathmin1 protein levels, which was positively verified in GBC tissues. The phosphorylation of four serine residues plays important roles on the microtubule-destabilizing activity of stathmin1 [4, 5]. Whether K9 acetylation had an effect on the activity of stathmin1? ERBB2 S310F mutation often occurs and activates MAPK/ERK pathway in GBC [2], which could promote ser25 phosphorylation of stathmin1 [4]. Our results suggested that K9 acetylation could decrease ser25 phosphorylation level of stathmin1 to promote the combination of stathmin1 and tubulin. Moreover, a MEK inhibitor could also reduce ser25 phosphorylation of stathmin1 to promote the combination stathmin1 with tubulin. These suggested that K9 acetylation could promote the microtubule-destabilizing activity of stathmin1.

Protein lysine acetylation is a reversible process regulated by acetyltransferases and deacetylases [11–13]. We firstly explored the acetyltransferases. According to previous studies, we co-transfected the PCAF-HA, CBP-HA, GCN5-HA and P300-HA with stathmin1-Flag plasmids to 293 T cells and confirmed that K9 acetylation of stathmin1 was regulated by PCAF. K9 acetylation of stathmin1 by PCAF was further verified in GBC cells. PCAF enhanced stathmin1 protein stability and the interaction of stathmin1 with tubulin through the promotion of K9 acetylation. We next analyzed the deacetylase. Results from NAM treatment indicated the deacetylation of stathmin1 was regulated by SIRT family. According to the localization of seven SIRT family members [14, 18], we co-transfected the sirt1-HA, sirt2-HA, sirt3-HA, sirt5-HA and stathmin1-Flag plasmids to 293 T cells and confirmed deacetylation of stathmin1 was regulated by sirt2. We also further verified that the deacetylation of stathmin1 was regulated by sirt2 in GBC cells, which decreased stathmin1 protein stability and the interaction of stathmin1 with tubulin. How did K9 acetylation inhibit the ubiquitination degradation of stathmin1? It has indicated an E3 ligase, RLIM could regulate the stability of stathmin1 protein [20, 21]. We confirmed that K9 acetylation inhibited the degradation of stathmin1 through disturbing the combination of acetylated stathmin1 and RLIM.

Cell migration is a polarized cellular process involving the cytoskeletal systems rearrangement conferring the metastatic properties of cancers [28–31]. The microtubule regulation viability of stathmin1 indicated its roles on cell migration, which is also verified in several studies [32–36]. K9 acetylation enhanced the activity of stathmin1 to promote GBC metastasis in vivo. Moreover, overexpression of K9 acetylation and stathmi1 protein expression was confirmed in GBC tissues. Altogether, K9 acetylation of stathmin1 by PCAF inhibited ubiquitination degradation of stahmin1, but promoted its microtubule-destabilizing activity, which caused gallbladder cancer metastasis (Fig. 8o). Our study revealed the underlying mechanism of K9 acetylation of stathmin1 and provided a potential target for GBC therapy.

## Materials and methods

### Cell culture

Human GBC-SD and 293 T cells were purchased from the Type Culture Collection of the Chinese Academy of Sciences (Shanghai, China). Human SGC-996 cells were obtained from Professor Yang’s Lab of Tongji University School of Medicine. Cells were cultured in DMEM or 1640 medium with 10% fetal bovine serum, 100 U/ml penicillin and 100 µg/ml streptomycin at 37 °C.

### Antibodies and reagents

Flag tag antibody (Cat. #AE063), HA tag antibody (Cat. #AE008), phospho-STMN1-S25 antibody (Cat. #AP0220), MYC tag antibody (Cat. # AE070) and ubiquitin antibody (Cat. #A19686) were purchased from Abclonal. Beta-tubulin (Cat. #2146), stathmin1 antibody (Cat. #3352), acetylated lysine antibody (Cat. #9441), phospho-ERK1/2 antibody (Cat. #4370), ERK1/2 antibody (Cat. #4695) and actin antibody (Cat. #3700) were purchased from Cell Signaling Technology. TSA (Cat. #HY-15144), NAM (Cat. # HY-B0150), MG132 (Cat. # HY-13259) and Protein A/G (Cat. # HY-K0202) were purchased form MedChemExpress. CHX (Cat. # S7418) was purchased from Selleck. Flag agarose beads (Cat. #B23102) was purchased from Bimake. HA magnetic beads (Cat. #88836) and MYC magnetic beads (Cat. #88842) were purchased from ThermoFisher Scientific. Binimetinib (Cat. #T2508) was purchase form Topscience.

### Preparation of stathmin1 K9 acetylated antibody

The antibody specifically recognizing K9 acetylation of stathmin1 was prepared by Shanghai Hui-Ou Biotechnology Co. Ltd (Shanghai, China). Briefly, the polypeptides of ASSDIQVK(Ac)ELEKRAS were firstly synthesized, then coupled with KLH as antigen to immunize rabbit. Anti-serum was collected after five doses of immunization. Antibody was purified in two steps. Step1, antigen affinity purification via non-acetylated peptides for serum to get the non-acetylated antibody; step2, antigen affinity purification by acetylated peptides for step1’s fluid to get the acetylated antibody. The specificity was analyzed through western blot and immunohistochemical staining detection.

### GBC samples

The GBC samples including frozen and paraffin-embedded tissues were from the tissue bank at the department of pathology Zhongshan Hospital affiliated with Fudan University. The study was carried out in accordance with the World Medical Association Declaration of Helsinki. Prior patient consent and approval from the Institutional Research Ethics Committee were obtained.

### siRNAs transfection

RNAi experiment was performed using commercially synthetic siRNA oligonucleotides. The siRNAs of RLIM, PCAF and sirt2 were following. si-PCAF: GCAGAUACCAAACAAGUUUAU, si-sirt2: GAGGCCAUCUUUGA GAUCATT and si-NC: UUCUCCGAACGUGUCACGUTT. Cells were seeded to 6-well culture dish and transfected at 60% density for 48 h using Lipofectamine^TM^2000 transfection reagent according to the manufacturer’s instructions (Cat. #11668027, Thermofisher). Briefly, before siRNA transfection, the medium was changed by serum-free DMEM. Then 5μl siRNA (20 μm) was mixed with 250μl serum-free DMEM for 5 min, and 5μl lipo2000 was mixed with 250μl serum-free DMEM for 5 min. The siRNAs solution was mixed with lipo2000 solution for 20 min, then added to cells. Cells medium was changed to high glucose DMEM with 10% serum after 6 h. Knockdown efficiency was detected through western blot after 48 h after transfection.

### Western blot

After treatment, cells were washed by PBS, then lysed by 0.3% NP40 buffer with protease inhibitor (G2008, Servicebio) and phosphatase inhibitor (G2007, Servicebio). Protein concentration was assayed using BCA protein assay kit (Cat. #23227, Thermofisher), Samples with 30 μg total protein were separated 12% SDS-PAGE electrophoresis, transferred to PVDF membrane, blocked by 5% non-fat milk solution, incubated with primary antibody overnight at 4 °C, then washed by PBST, incubated HRP-conjugated secondary antibody, then washed by PBST. Finally, the target protein was visualized through an enhanced chemiluminescence system.

### Co-immunoprecipitation and immunoprecipitation

Co-immunoprecipitation and immunoprecipitation were performed as described previously [37]. Briefly, 293 T cells at 60% confluence were transiently transfected with plasmids for 48 h, then lysed with 0.3% NP40 for immunoprecipitation or 0.1% NP40 for co-immunoprecipitation with 1 mM PMSF and 1 mM Na_3_VO_4_. Lysis buffer was precleaned by IgG, then incubated with HA beads, MYC beads or Flag beads overnight at 4 °C. The aggregates were centrifuged at 20,000 g for 10 min, washed three times, then mixed with an equal volume of 2*SDS sample buffer to preparing immunoblotting sample.

### Migration and invasion assay

GBC-SD and SGC-996 stable cells expressed wild type and K9R mutant stathmin1 were suspended at serum-free DMEM medium, then 100 μl of cell suspension was seeded to the upper chamber of transwell (Cat. #3422, Costar), the lower chamber was added with 600 μl DMEM medium with 10% FBS to culture for 18 h. The chamber was fixed with methyl alcohol, stained by crystal violet buffer. The number of migrated and invaded cells were counted from five random fields.

### Immumohistochemical staining

Immunohistochemistry (IHC) was performed as previously described [38]. IHC results were scored according to percentage of positive cells and intensity of staining. The percentage of positive cells was divided into five grades: less than 10% as grade 0, 10–25% as grade 1, 25–50% as grade 2, 50–75% as grade 3, and more than 75% as grade 4. The intensity of staining was divided into four grades: negative staining as grade 0, light brown as grade 1, brown as grade 2, and heavy brown as grade 3. IHC staining scores were elevated: 0 score, negative (−); 1–4 scores, weakly positive (+); 5–8 scores, moderately positive (++); 9–12 scores, strongly positive (+++).

### GBC lung metastasis model

Animal studies were carried out in accordance with the approved guidelines of Animal Ethical Committee of Zhongshan Hospital affiliated with Fudan University. Nude mice (male or female, Bablc, SLAC, Shanghai) were at 4–5 weeks. SGC-996 stable cells expressed wild type and K9R mutant stathmin1 were suspended at PBS with a concentration of 3*10^7^/ml, then 100μl of cell suspension was injected to six nude mice of one random group through tail vein. The growth of metastatic tumor volume was monitored through Tanon ABL X5 Live Animal Imaging System every two weeks. Mice were intraperitoneally injected with 100μl of d-luciferin-potassium salt buffer (30 mg/ml), then anesthetized. Living imaging was performed at 10–20 min after injection.

### Statistics

Statistical analysis was performed for differences with one-way analysis of variance (ANOVA) in more than two groups and two-tailed student’s *t*-test in two groups. Statistical results were shown as the mean ± SD from at least three times experiments. Differences were considered to be significant when *p < 0.05.

## Supplementary information

Figure S1

Dataset 1

## Data Availability

All the data used during the study are available from the corresponding author on request.
