# Supplementary figures and images for: Acetylation stabilizes stathmin1 and promotes its activity contributing to gallbladder cancer metastasis
Source: Cell Death Discov. 2022 May 17;8:265. doi: 10.1038/s41420-022-01051-z (PMC9114396; doi:10.1038/s41420-022-01051-z)

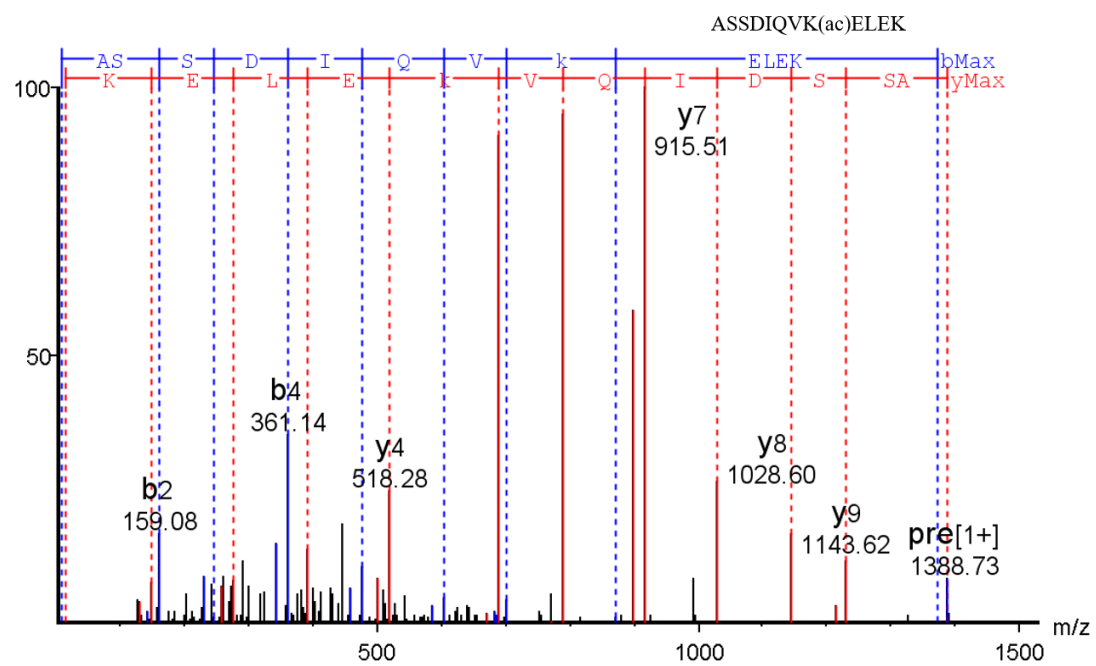

Figure S1. The mass spectrum graph of K9 acetylated peptides of stathmin1.

Supplement: Supplementary file 1 — Figure S1 [file 41420_2022_1051_MOESM1_ESM.pdf]
